# Total flavonoids of Astragalus protects glomerular filtration barrier in diabetic kidney disease

**DOI:** 10.1186/s13020-024-00903-3

**Published:** 2024-02-16

**Authors:** Pei-Yu Liu, Kin-Fong Hong, Ya-Di Liu, Zhong-Yan Sun, Ting-Ting Zhao, Xu-Ling Li, Chi-Chou Lao, Shu-Feng Tan, Hai-Ying Zhang, Yong-Hua Zhao, Ying Xie, You-Hua Xu

**Affiliations:** 1 Faculty of Chinese Medicine, State Key Laboratory of Quality Research in Chinese Medicine, Macau University of Science and Technology, Taipa, Macao People’s Republic of China; 2 Institute of Chinese Medical Sciences, State Key Laboratory of Quality Research in Chinese Medicine, University of Macau, Macao, China; 3 State Key Laboratory of Traditional Chinese Medicine Syndrome, The Second Affiliated Hospital of Guangzhou University of Chinese Medicine, Guangzhou, Guangdong China

**Keywords:** Diabetic kidney disease, Flavonoids, Astragalus, Proteinuria

## Abstract

**Background:**

Diabetic kidney disease (DKD) is a prevalent complication of diabetes and the leading cause of end-stage renal disease. Recent evidence suggests that total flavonoids of Astragalus (TFA) has promising effects on diabetes; however, its influence on DKD and the underlying mechanism remains unclear.

**Methods:**

In this study, we induced the DKD model using streptozotocin (STZ) in male C57BL/6J mice and utilized glomerular endothelial cell (GEC) lines for in vitro investigations. We constructed a network pharmacology analysis to understand the mechanism of TFA in DKD. The mechanism of TFA action on DKD was investigated through Western blot analysis and multi-immunological methods.

**Results:**

Our findings revealed that TFA significantly reduced levels of urinary albumin (ALB). Network pharmacology and intracellular pathway experiments indicated the crucial involvement of the PI3K/AKT signaling pathway in mediating these effects. In vitro experiments showed that TFA can preserve the integrity of the glomerular filtration barrier by inhibiting the expression of inflammatory factors TNF-alpha and IL-8, reducing oxidative stress.

**Conclusion:**

Our findings demonstrated that TFA can ameliorates the progression of DKD by ameliorating renal fibrosis and preserving the integrity of the kidney filtration barrier. These results provide pharmacological evidence supporting the use of TFA in the treatment of kidney diseases.

**Supplementary Information:**

The online version contains supplementary material available at 10.1186/s13020-024-00903-3.

## Introduction

Diabetic kidney disease (DKD) is a chronic microvascular complication in individuals with type 1 or 2 diabetes, associated with multiple deviations from normal homeostasis, could cause significant changes in blood flow and metabolism, which in turn activate various pathways in the kidney. The kidney glomerular filtration barrier, which consists of the fenestrated glomerular endothelium, the glomerular basement membrane, podocytes, and the slit diaphragm between the podocytes, plays a vital role in preventing the passage of albumin and blood cells into urine. In the early stages of DKD, the disease primarily affects the glomeruli. Alterations in renal endothelial cells play a significant role in initiating and advancing DKD [1]. Changes in the glycocalyx of glomerular endothelial cells, the loss of these cells, and the reduction in capillary density contribute to the decline in glomerular filtration function, eventually leading to end-stage renal disease. It is found that high glucose levels can induce micro-inflammation and increase the risk of damage to glomerular endothelial cells. Inflammation has been recognized as a key factor in DKD development; oxidative stress (OS), transcription factors such as nuclear factor κB (NF-κB), janus kinase/signal transducers and activators of transcription (JAK/STAT) pathway were believed to play pivotal role in it [2–4]. Therefore, therapeutic strategies for diabetic kidney disease focus on inhibiting glomerular endothelial cell as well as alleviating micro-inflammation are believed to have beneficial effects against progression of DKD.

Besides physical barrier, glycocalyx on the surface of glomecular endothelial cells also play a role in preventing proteinuria in DKD. Glycocalyx is the polysaccharide-protein complex layer on the luminal side of vascular endothelial cells. The molecular sieve effect of the glycocalyx structure determines the permeability of glomerular blood vessels, and the negative charge nature of the glycocalyx also makes the blood vessels act as a charge barrier [5]. Under physiological conditions, its main function is to regulate vascular endothelial permeability and the interaction between blood cells and endothelial cells. Under inflammatory conditions, a variety of inflammatory mediators cause the shedding of the glycocalyx of the vascular endothelium, weakening its vascular protective function [6]. At the same time, heparan sulfate, a component of the vascular endothelial glycocalyx, can regulate the development of inflammation, including regulating the migration of leukocytes on the side of the vascular lumen and their tight adhesion to endothelial cells, regulating the transport of chemokines from tissues to the vascular lumen, etc. [7, 8].

There is a wealth of research exploring the effects of Astragalus (known as Huang Qi in Chinese) on diabetes. Astragalus Radix is rich in active components such as polysaccharides, astragalosides, and flavones. In recent investigations, we have discovered that oral administration of Astragalus Radix extract can enhance the integrity of both the gut barrier and the blood–brain barrier [9, 10]. These findings have demonstrated positive effects in inhibiting complications associated with diabetes. Besides that, studies have demonstrated that total flavonoids of Astragalus (TFA) can impact gut barrier function and inflammatory markers by exhibiting anti-inflammatory properties or enhancing the expression of junction proteins in intestinal epithelial cells [11, 12]. Previous research has shown the protective effects of TFA on diabetes and diabetic nephropathy in mice by eliciting an anti-inflammatory response and modulating the NF-ΚB and MAPK signaling pathways in glomerular mesangial cells [11, 13]. However, the influence of TFA on the glomerular filtration barrier remains unclear. In this study, we aimed to investigate the impact of TFA on the expression of filtration barrier-related proteins in glomerular endothelial cells and podocytes, as well as its role in alleviating albuminuria and up-regulating tight junction proteins and glycocalyx in DKD mice.

## Materials and methods

### Materials

Human renal Glomerular endothelial cells were obtained from ScienCell (San Diego, USA). Total flavonoids of Astragalus used in this study was obtained from Chengdu Pusi Biotechnology, Co., Ltd. (Chengdu, Sichuan, China). Metformin was purchased from GBCBIO Technology (Guangzhou, Guangdong, China). Glucose was purchased from Chemical Reagent Factory (Guangzhou, China). Insulin was provided by Yuanye Bio-Technology Co., Ltd. (Shanghai, China). Cell Counting Kit-8 (CCK-8) was obtained from Dojindo Molecular Technologies, Gaithersburg (MD, USA). Detection kits for MDA (S0131) and SOD (S0101S) were derived from Beyotime (Shanghai, China). Detection kits for BUN (C013-2-1), Cr (C011-2-1), and mALB (E038-1-1) were provided by Jiancheng (Nanjing, China). BCA (PC0020), RIPA (R0020-100 ml) were purchased from Solarbio Science & Technology Co., Ltd. (Beijing, China). Antibodies for Beta Actin (66009-1-Ig), AKT (60203-2-Ig), Phospho-AKT (Ser473), FIS1 (66635-1-Ig), HSP60 (15282-1-AP), TNF-alpha (60291-1-Ig), IL-1Beta (16806-1-AP) were purchased from Proteintech (Boston, MA, USA). Antibodies for Phospho-NF-κB p65 (Ser536) (93H1) (3033T) and NF-κB p65 (D14E12) (8242T) were purchased from Cell Signal Technology (Boston, MA, USA). Antibody for PI3 Kinase p110 beta (bs-10657) was abtained from Bioss (Beijing, China), and goat Anti-Rabbit IgG (H + L) HRP (S0001) were purchased from Affinity (OH, USA). Antibodies for CD138/SDC1/Syndecan-1 (DL-101) (sc-12765) and glypican-1 (A-10) (sc-365000) were obtained from Santa Cruz Biotechnology Inc. (Santa Cruz, CA, USA). Occludin were bought from Proteintech (California, USA). Goat anti-Rabbit IgG (H + L) secondary antibody FITC (65-6111), goat anti-Mouse IgG (H + L) secondary antibody FITC (62-6511), DAPI, and dilactate (2445405) were obtained from Invitrogen (Carlsbad, CA, USA). TFA (purity: ≥ 90.0%) was bought from Pusi Biotecnology (Chengdu, China), and its quality was verified according to our previously reported method [9].

### Cell lines and cell culture

Glomerular endothelial cells (GECs) were derived from ScienceCell (Fenghui Biological, China). Cells were cultured in MEM medium (Gibco) supplemented with 10% fetal bovine serum, 100 U/mL penicillin, and 100 mg/mL streptomycin at 37 °C in a 5% CO_2_ incubator.

### Animal studies

**Fig. 1:**
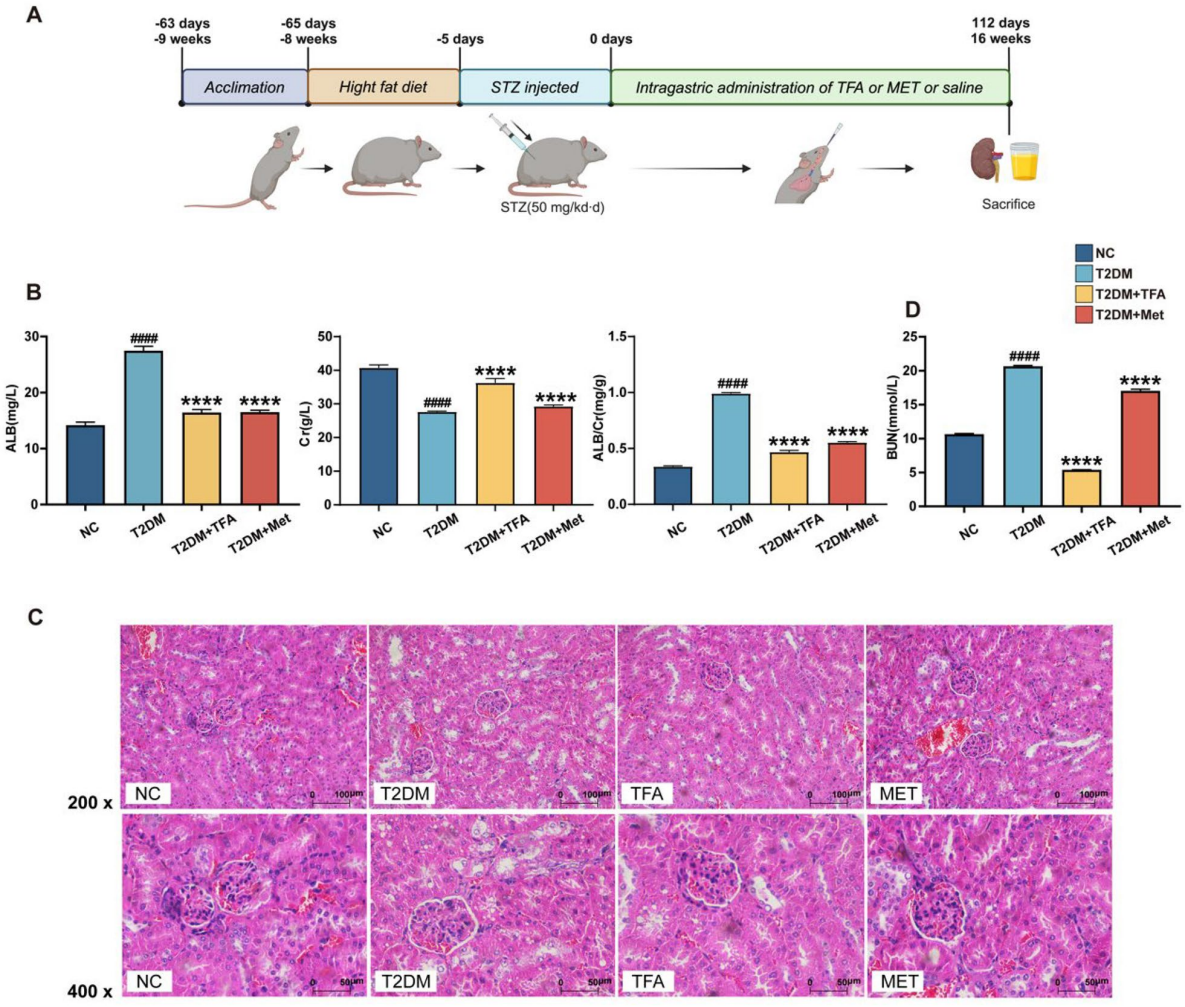
TFA Attenuated DKD in Diabetic Mice. **A** Timeline of the administration in each group mouse. **B** Effect of TFA on biochemical parameters in DKD mice. **C** H&E staining of TFA in different groups on DKD. *NC* natural control, *T2DM* type 2 diabetes mellitus, *TFA* total flavonoids of Astragalus, *MET* metformin. Values are presented as means ± SD. ^**#**^p < 0.05, ^**##**^p < 0.01, ^**###**^p < 0.001, ^**####**^p < 0.0001, vs. NC. *p < 0.05, **p < 0.01, ***p < 0.001, ****p < 0.0001, vs. T2DM

Ethical approval for all animal care and experimental protocols was obtained from Macau University of Science and Technology. All animal care and operations were according to the guidelines of the U.S. National Institutes of Health in the care and use of Laboratory Animals (NIH publication No. 85-23, revised 1996). 8–10 weeks old, 20 ± 5 g male C57BL/6 J mice were bought from Guangdong Medical Laboratory Animal Center and were housed in a specific pathogen-free (SPF) environment. To induce the DKD model, streptozotocin (STZ) was administered intraperitoneally at 25 mg/kg for five days (Fig. 1A). The model mice were provided with a high-fat diet, while the control group was fed a regular diet. Mice with a blood glucose level exceeding 11.1 mmol/L were selected for further experiments. Each group consists of six mice: (1) T2DM group (HFD, High fat diet); (2) TFA group (TFA group, HFD, TFA 5 mg/kg/d) [9]; and (3) positive control group (HFD, metformin, 0.15 g/kg/d). All drugs were orally administrated for 16 weeks. At the end of the experiment, mice were killed by the cervical dislocation method; the serum and urine were collected for further study.

### Hematoxylin–eosin staining

The kidneys were collected after rinsing with normal saline. A portion of the kidney tissues was fixed in 10% neutral formaldehyde, embedded in paraffin, and utilized to create pathological sections. Hematoxylin–eosin (H&E) staining was carried out using a standard procedure.

### Western blot analysis

Protein extraction from kidney tissues and GECs was subjected to Western Blot analysis. The proteins were extracted by RIPA electro-transferred onto a PVDF membrane and incubated overnight at 4 °C with primary antibodies. The membranes would exposed to secondary antibodies at room temperature for 1 h after washing the membranes with TBST. The densitometric analysis of the protein blots was performed using ImageJ software.

### Network pharmacology

#### Database and analysis software

Database and Analysis Software Databases including TCMSP (http://lsp.nwu.edu.cn/tcmsp.php), Genecards (http://www.genecards.org), UniProt (https://www.uniprot.org), String (https://string-db.org), Bioconductor (http://www.bioconductor.org), Enrich (https://maayanlab.cloud/Enrichr/enrich), and DAVID (https://david.ncifcrf.gov/) were applied to collect data. Cytospase 3.8.0 software was used to analyze the data.

#### Collection and screening of chemical components

The candidate compounds of Astragalus were obtained from the TCMSP analysis platform. This platform utilizes absorption, distribution, metabolism, and excretion (ADME) related indexes to analyze the compounds and assist in screening active compounds. We require that the candidate components meet two of the following parameters: (1) Oral bioavailability (OB) ≥ 30%, and (2) Drug-likeness (DL) ≥ 0.18 [14, 15].

#### Target collection and network construction and protein interaction analysis

We combined the target of TFA and the target of diabetes to obtain potential targets and plotted this relationship into a network by String. Then, import the information of node and combined score (combined score) into Cytoscape to draw the interaction network, and analyze the network to obtain the PPI network [16].

#### Gene ontology term enrichment and Kyoto encyclopedia of genes and genomes analysis

In order to study the synergistic effect of TFA in ameliorating DKD at the signal pathway level, we performed analyzed the Gene Ontology Term Enrichment (GO) and the Kyoto Encyclopedia of Genes and Genomes (KEGG) by BioConduntor and R Language to reveal the mechanism of action of TFA. GO analysis was performed using ID correspondence or sequence annotation methods.

### Immunofluorescence

After a 24 h incubation with drugs, cells were fixed using a 4% paraformaldehyde solution. A 0.1% Triton X-100 PBS solution was applied to the cells for 10 min at room temperature to enhance permeabilization. Subsequently, the cells were washed with PBS and blocked to prevent unspecific binding by incubating them with a blocking buffer (5% BSA in PBS) for 60 min at room temperature. Following the blocking step, the cells were exposed to primary antibodies overnight at 4 °C, followed by a secondary antibody for 1 h in the dark at room temperature. Nuclei were counterstained with DAPI. Images were acquired using a confocal microscope (Leica TCS SP8, Germany) under standardized conditions and analyzed using ImageJ software.

### Statistical analysis

Figure preparation and statistical analysis were carried out using GraphPad Prism 8.0 software (GraphPad Software Inc., CA, USA). Fluorescent images were processed with the open-source software ImageJ. All data were obtained from more than three independent repeated experiments. All data fitting into the normal distribution were expressed as mean ± standard deviation (SD), and the difference among groups was analyzed by one-way ANOVA. Significance was accepted at *p* < 0.05 or less.

## Results

### TFA attenuated DKD in diabetic mice

Blood urea nitrogen (BUN) and creatinine (Cr) are well-established indicators of kidney function. To investigate the metabolic changes induced by the drug in DKD mice, we conducted a metabolite analysis of urine from mice receiving TFA. We found diabetic mice exhibited significantly increased 24-h urinary albumin excretion and urine Cr compared to control mice (Fig. 1B), and treatment with TFA led to a remarkable reduction in 24-h urinary albumin excretion and urine Cr, and this effect persisted throughout the TFA treatment period. Additionally, the serum levels of BUN were notably elevated in DKD mice, and TFA administration effectively suppressed this increase. Collectively, these findings highlight the significant inhibition of DKD progression achieved through TFA treatment.

In order to validate the effects of TFA in mitigating DKD damage, H&E staining was performed on kidney tissues. The result illustrated the noticeable presence of glomerular basement membrane thickening and glomerular sclerosis in DKD mice (Fig. 1C). And oral administration of TFA exhibited a significant preservation of nephron histological integrity and inhibited glomerular sclerosis.

### TFA ameliorated oxidative stress and inflammation in vitro

**Fig. 2:**
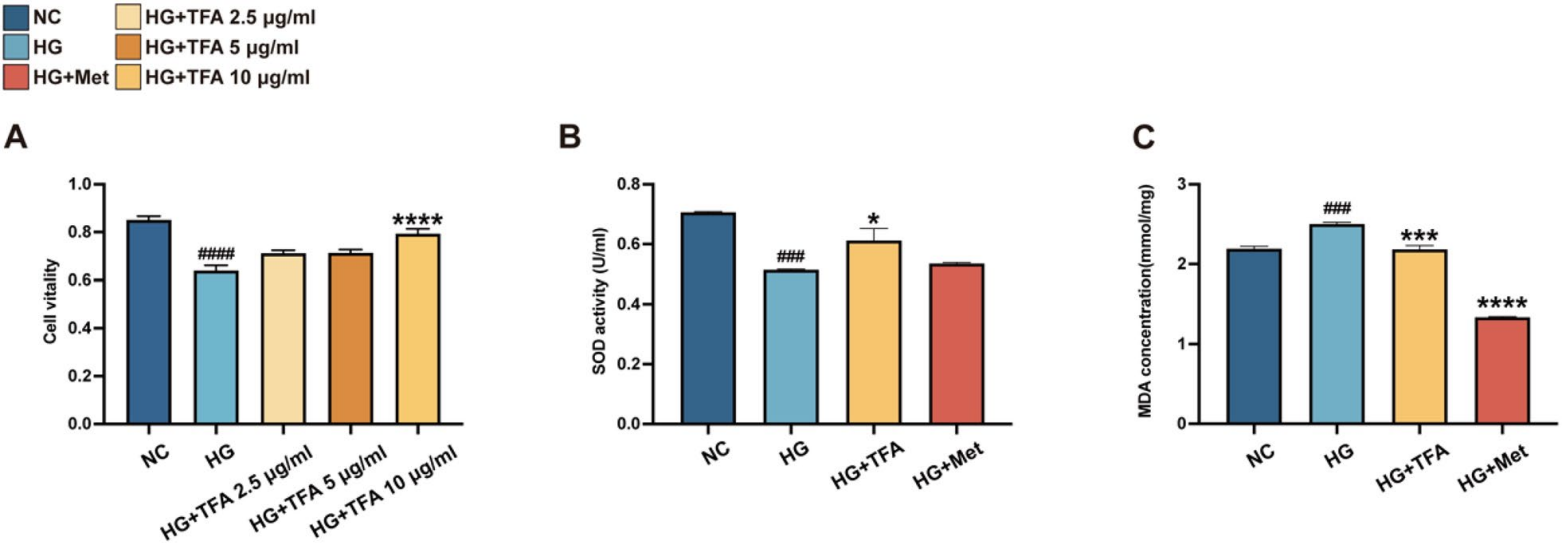
TFA increased cell viability and ameliorated oxidative stress in GECs. ^**###**^p < 0.001, and ^**####**^p < 0.0001, vs. NC. *p < 0.05, ***p < 0.001, and ****p < 0.0001, vs. HG

In order to investigate direct effect of TFA on glomerular endothelial cells, GECs cell were applied. We firstly determined the optimal concentration of TFA on high glucose (HG, 30 mM) induced GECs cells using the CCK-8 assay (Fig. 2A), and finally 10 μg/mL of TFA was used in the following study. Superoxide dismutase (SOD) has been found to have antagonistic effect on reactive oxygen species (ROS) and helps prevent damage to body tissues and related health conditions [17, 18]. In this study, we measured both SOD and malondialdehyde (MDA) levels to evaluate the impact of TFA on cellular OS comprehensively. Our findings revealed that the SOD value significantly decreased in GECs incubated with high glucose compared with the normal group, and TFA effectively reversed the impairment of the cell's antioxidative ability (Fig. 2B and C), the level of the antioxidant enzyme SOD was markedly decreased, while the MDA content was increased.

**Fig. 3:**
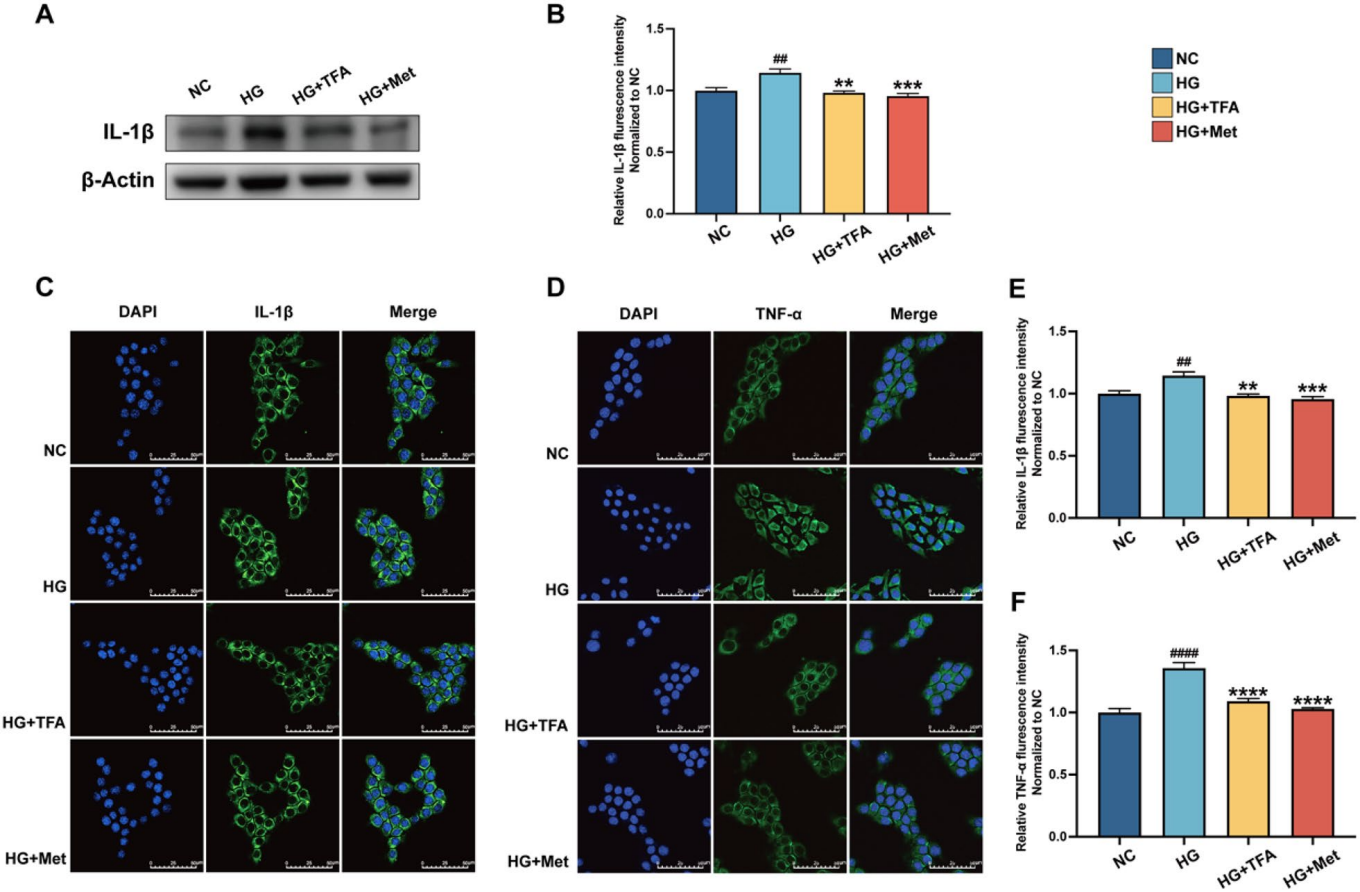
TFA decreases high glucose-induced IL-1β and TNF-α release in GECs. Western Blot (**A**, **B**) and Immunofluorescence assay (**C**–**F**, magnification:×800) for IL-1β and TNF-α. ^**#**^p < 0.05, ^**##**^p < 0.01, and ^**####**^p < 0.0001, vs. NC. *p < 0.05, **p < 0.01, ***p < 0.001, ****p < 0.0001, vs. HG

DKD is considered as an inflammatory disease caused by glucose and lipid metabolism disorders. Studies have provided evidence that patients or animal models of DKD exhibit increased release of inflammatory cytokines. The elevated inflammatory cytokines have been found to promote macrophage infiltration, enhance glomerular basement membrane formation and degradation, endothelial glycocalyx damage, and worsen renal tubular fibrosis, contributing to accelerated glomerular sclerosis [19–21]. In our previous animal study, TFA oral administration reduced serum IL-1β and TNF-α levels in DKD animals [9]. To further determine the role of TFA anti-inflammation in vitro, the GECs were incubated with 30 mM glucose for 24 h and detected the level of IL-1β and TNF-α. The results showed that TFA incubation could significantly reduce the level of IL-1β and TNF-α (Fig. 3).

### TFA reversed syndecan-1 and glypican-1 decrease

**Fig. 4:**
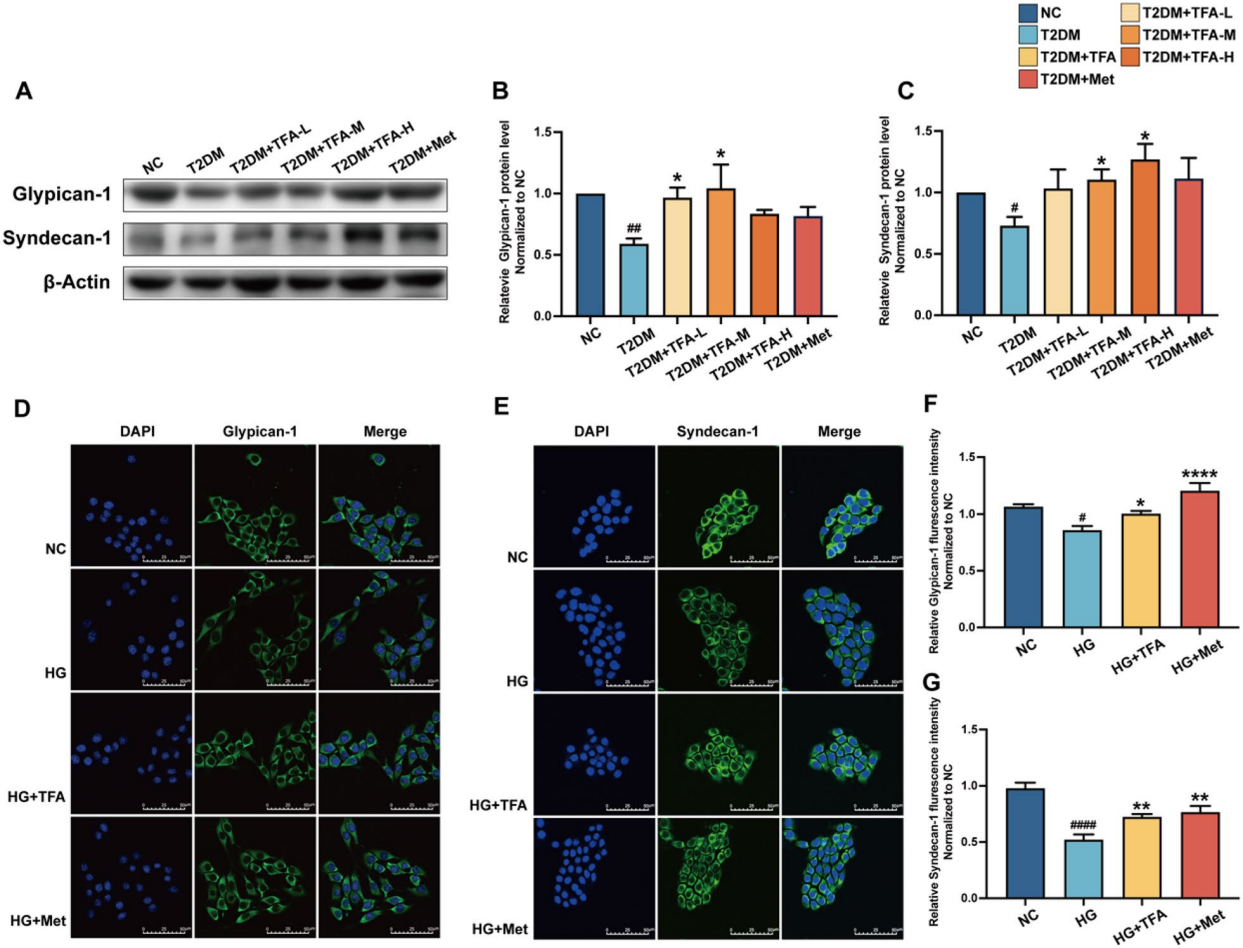
TFA induces expression syndecan-1 and glypican-1 in GECs. Western blot analysis of syndecan-1 and glypican-1 (**A**–**C**), TFA‑L: 5 mg/kg/d; TFA‑M: 25 mg/kg/d; TFA‑H: 50 mg/kg/d. Immunofluorescence assay for glypican-1 and syndecan-1 under confocal microscope (**D**–**G**) (magnification:×800). ^**#**^p < 0.05, ^**##**^p < 0.01, and ^**####**^p < 0.0001, vs. NC. *p < 0.05, **p < 0.01, and ****p < 0.0001, vs. HG

Inflammatory cytokines have an important impact on glycocalyx damage, so we examined changes in syndecan-1 and glypican-1 in DKD. The syndecan-1 and glypican-1 are core proteins of the endothelial glycocalyx surrounding the endothelial cell membranes and plays a significant role in maintaining the integrity of the glomerular filtration barrier. Qiu and colleagues [22] showed that 30 mM glucose could decrease the expression of core protein syndecan-1 and glypican-1 of human GECs. In the present study, we determined the effect of TFA on the GECs in the high glucose model. As shown in Fig. 4, TFA or 1 mmol/L metformin increased the expression of glypican-1 and syndecan-1 compared with the HG. Therefore, we believe that TFA can increase the expression of syndecan-1 and glypican-1, thereby protecting the glomerular filtration barrier.

### TFA preserved the integrity of the glomerular filtration barrier

**Fig. 5:**
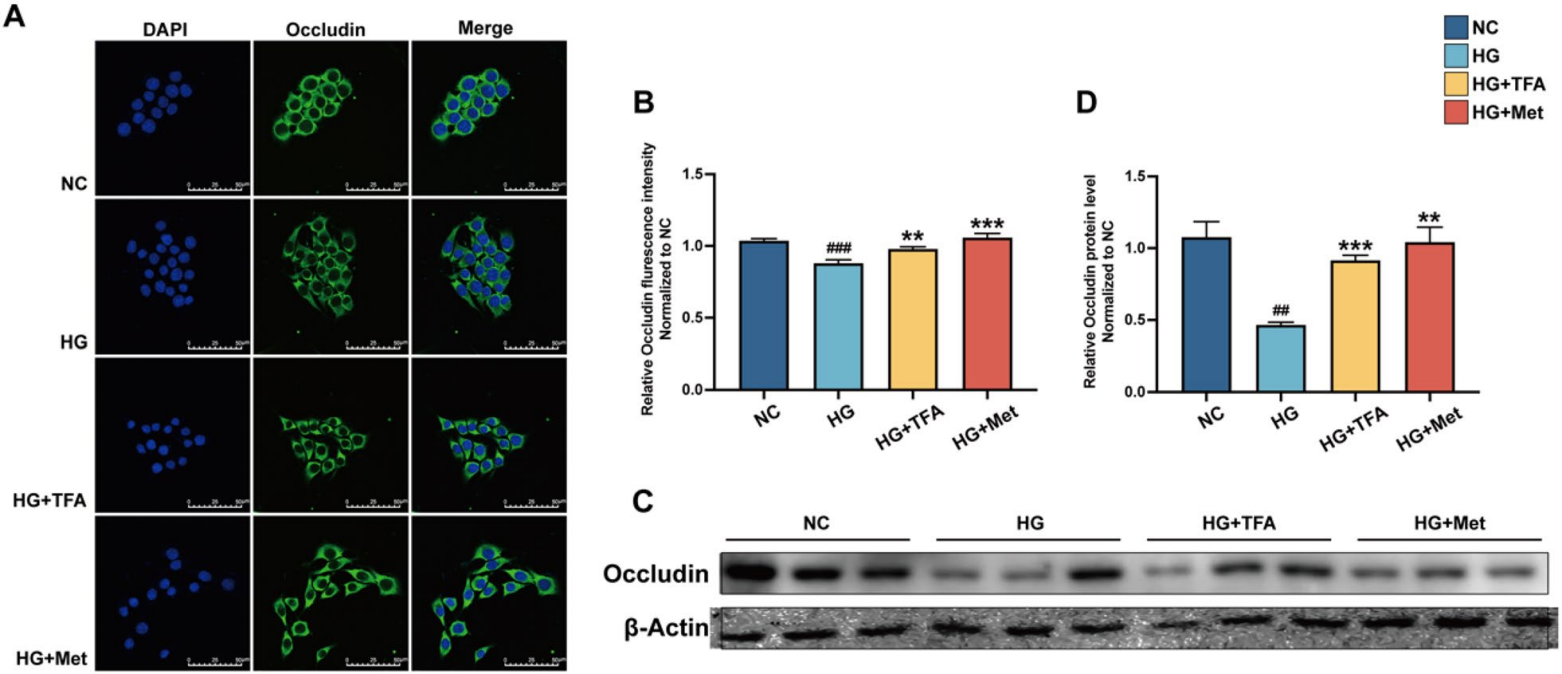
Effect of TFA on the glomerular filtration barrier in GECs. Immunofluorescence and western blot assay for GECs tight junction protein occluding. ^##^p < 0.01, and ^###^p < 0.001, vs. NC. ^*^p < 0.05, ^**^p < 0.01, and ^***^p < 0.001, vs. HG.

Preserving the glomerular filtration barrier's integrity is crucial in preventing proteinuria. Inflammatory cytokines can lead to a decrease in the expression of tight junction proteins [23], thereby damaging the glomerular filtration barrier [24]. In the present study, we observed (Fig. 5) that high glucose treatment significantly suppressed the expression of occludin in GECs, and treatment with TFA could significantly increase the cells' expression of tight junction proteins.

**Fig. 6:**
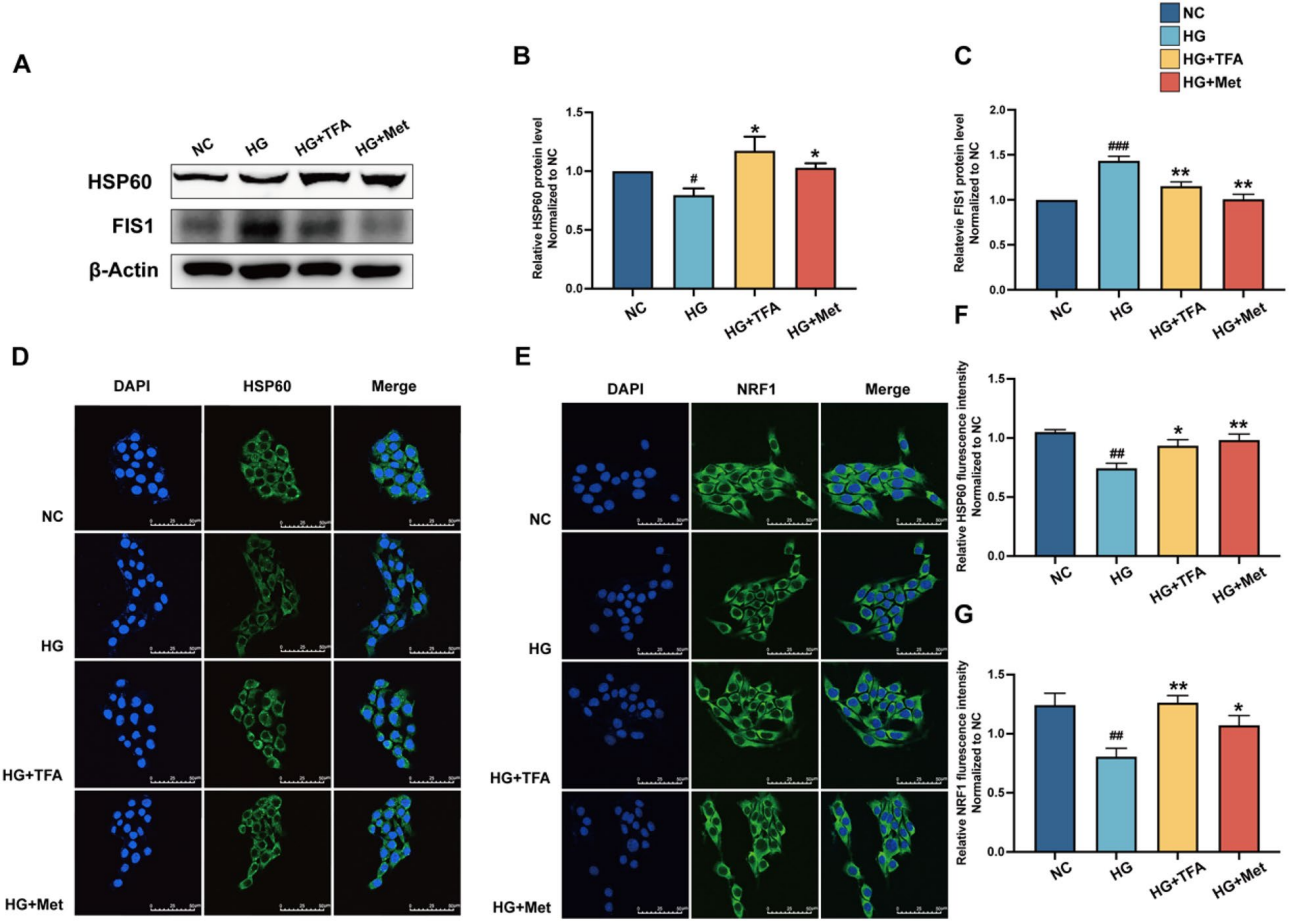
TFA promoted mitochondrial biogenesis and maintaining energy metabolic balance. **A** Western blot analysis of HSP60 and Fis1. Relative expression and activation of **B** HSP60 and **C** Fis1 were analyzed by ImageJ software. Immunofluorescence assay for HSP60 and Nrf1 (**D**, **E**) under a laser scanning confocal microscope and relative fluorescence intensity for them were determined (**F**, **G**) (magnification:×800). ^**#**^p < 0.05, ^**##**^p < 0.01, and ^**###**^p < 0.001, vs. NC. ^*^p < 0.05, ^**^p < 0.01, vs. HG

The integrity of the tight junctions in the glomerular filtration barrier relies on adequate energy supply, highlighting the importance of mitochondrial function. In endothelial cells derived from individuals with T2DM, mitochondrial networks exhibit fragmentation, accompanied by increased expression of mitochondrial fission protein 1 (Fis1). Conversely, overexpression of Fis1 in healthy blood vessels impairs vasodilation and increases mitochondrial superoxide production, suggesting a causative role [25]. Loss of Nrf1 in the presence of reactive oxygen species (ROS) results in significant oxidative stress damage [26]. HSP60, a core marker of the mitochondrial unfolded protein response (UPRmt), plays a crucial role in the cellular response to oxidative stress. In this study, we utilized Western Blot analysis to examine the expression of Fis1 and HSP60 (Fig. 6). We found the expression of Fis1 was up-regulated in the diabetes model group (p < 0.01, vs. NC), indicating mitochondrial damage; following treatment with TFA or metformin, the expression of Fis1 was significantly down-regulated; we further found a significant down-regulation of NRF1 and HSP60 in the diabetes model group, indicating continuous activation of UPRmt in GECs. Expectively, their expression levels were significantly upregulated following treatment with TFA or metformin. The above results suggest that TFA protected glomerular filtration barrier function by promoting mitochondrial biogenesis and maintaining energy metabolic balance.

### Network pharmacology analysis and differential metabolite analysis of urine

**Fig. 7:**
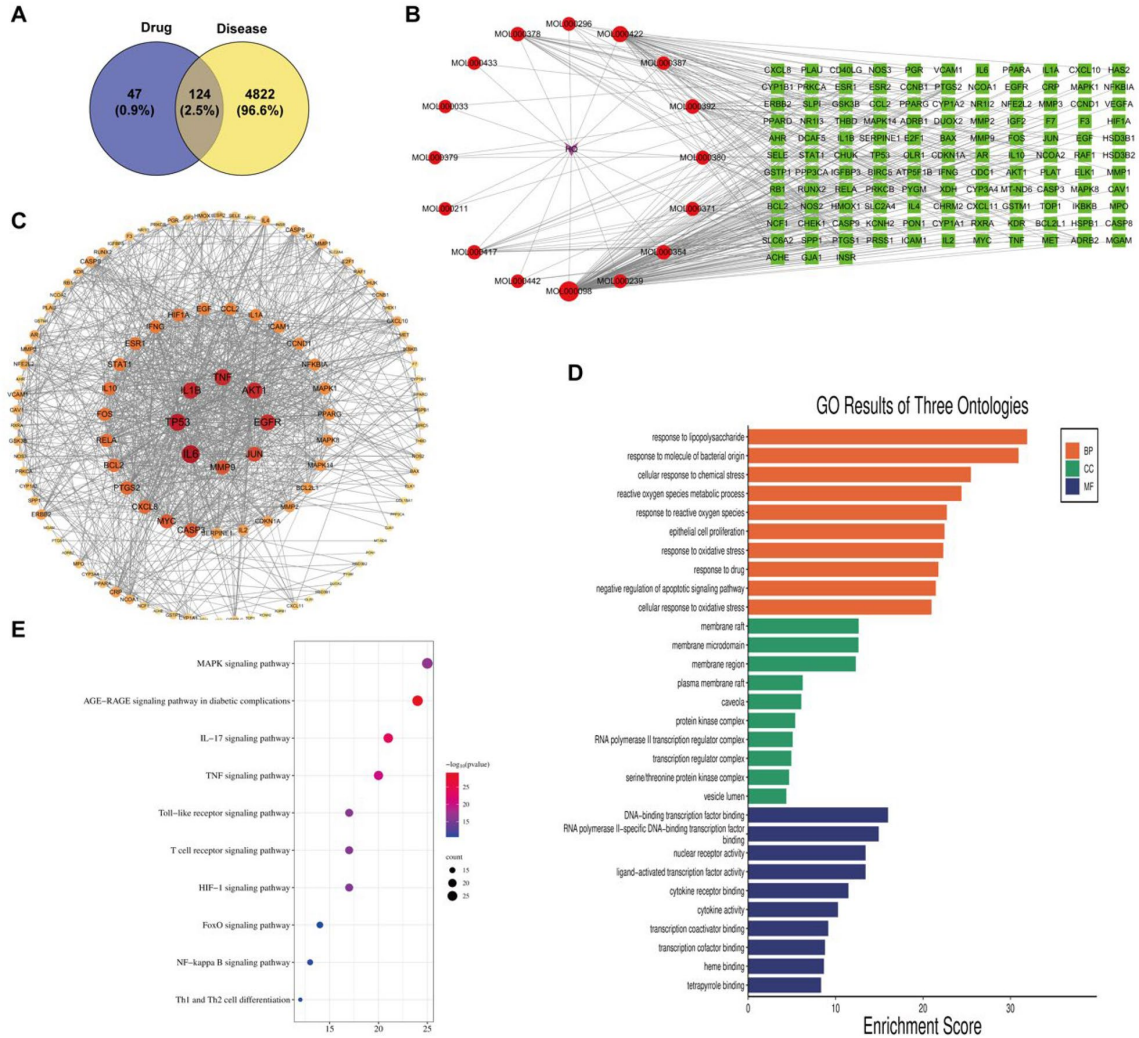
Network pharmacology analysis and differential metabolite analysis were performed on urine samples

In order to investigate the potential components and molecular targets of TFA on DKD, we performed a network pharmacological analysis. Our analysis revealed 17 compounds (see Additional file 1: Table S1) in TFA that could potentially have an active role. Additionally, we conducted a search for 4,946 DKD target genes. By creating a Venn diagram of the intersecting targets, we identified 124 common targets (Fig. 7A).

To comprehensively understand the mechanism of TFA in DKD, we constructed a network linking TFA-active components, their corresponding targets, and DKD-related genes (Fig. 7B). Through high-frequency node analysis in the PPI network (Fig. 7C), we identified IL-6 (interleukin 6), TP53, IL-1β (interleukin-β), TNF, AKT1, EGFR, JUN, CASP3 (caspase 3), MMP9, and MYC as central nodes in the network. GO analysis (Fig. 7D) and KEGG analysis (Fig. 7E) were conducted to enrich pathways and functions based on putative targets. The functional analysis revealed significant enrichment of the MAPK signaling pathway and the phosphatidyl-inositol 3-kinase/serine-threonine kinase (PI3K/AKT) signaling pathway in DKD-related pathways. Additionally, the functional analysis data indicated that these potential targets not only regulated cell proliferation, apoptosis, growth, and inflammatory response, but also influenced the modulation of the PI3K/AKT signaling pathways.

### TFA modulated PI3K/AKT pathway

**Fig. 8:**
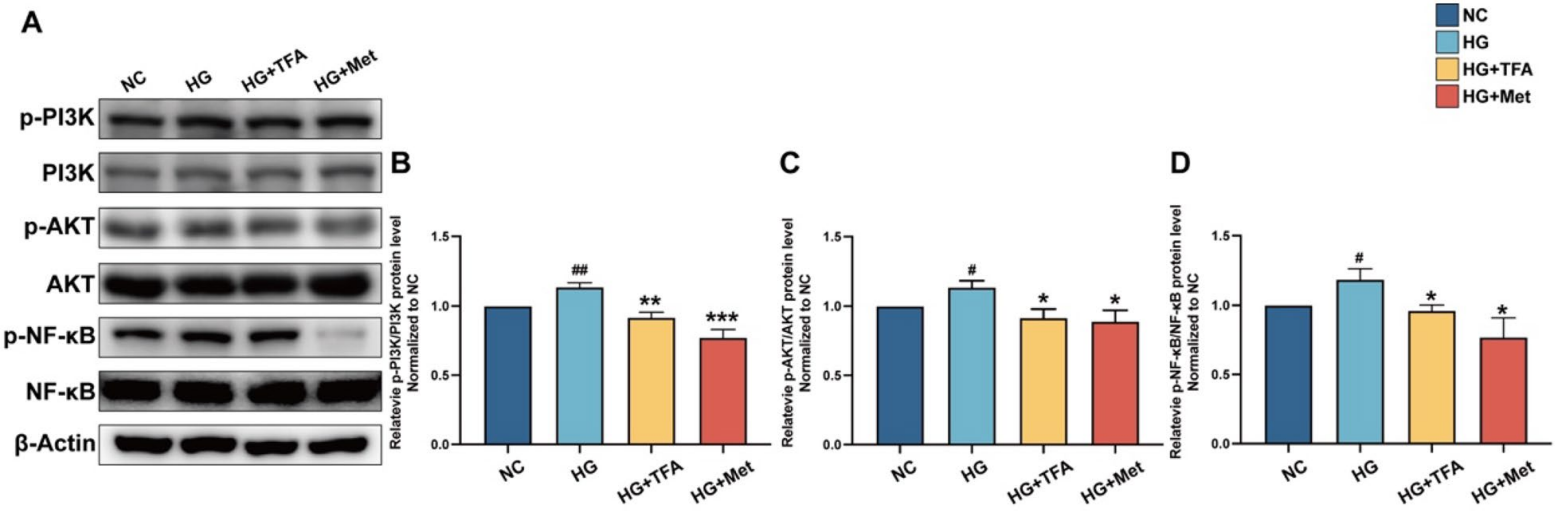
Western blot analysis of PI3K/AKT signaling pathways (**A**). Relative expression and activation of PI3K and AKT (**B**) were analyzed by the ImageJ software. Values are presented as means ± SD. ^**#**^p < 0.05, ^**##**^p < 0.01, vs. NC. *p < 0.05, **p < 0.01, ***p < 0.001, vs. HG

In order to verify the signaling pathway identified earlier as being influenced by TFA, the expression and activation of specific proteins were examined using Western Blot analysis. The results revealed that T2DM models of GECs exhibited significant activation of AKT and PI3K proteins, whereas TFA administration effectively reduced their phosphorylation levels (Fig. 8).

## Discussion

Diabetic kidney disease is one of the major complications for diabetic patients and the most significant cause of end-stage kidney disease [27]. Many conventional approaches, including renin–angiotensin–aldosterone system blockade, blood glucose level control, and body-weight reduction, often fail to yield satisfactory outcomes in clinical practice. The use of Chinese medicine for DKD has shown promising results and gained recognition globally [28]. In this study, we aimed to investigate TFA's effects and underlying mechanisms in inhibiting the progression of DKD.

Astragalus, a renowned traditional Chinese medicine, is commonly used in clinical practice for treating renal diseases. TFA was extracted from Astragalus and has been the subject of various studies, which have consistently demonstrated its efficacy in reducing proteinuria and improving kidney function in experimental models [11, 29, 30]. However, the exact underlying mechanism of these effects remains unclear. In our current study, we discovered that TFA administration significantly reduced BUN, Cr, and ALB levels. Furthermore, histological examination using H&E staining revealed that TFA preserved the structural integrity of both the glomerulus and the filtration barrier.

Proteinuria serves as the primary manifestation of DKD [31], while an elevated level of ALB is recognized as the initial characteristic of DKD [32]. Although the complete understanding of the underlying mechanism remains elusive, it is widely believed that the critical event involves alterations in the structure and function of the glomerular filtration membrane. The glomerular filtration membrane predominantly comprises glomerular endothelial cells and podocytes, both susceptible to damage under certain conditions [33]. Notably, the endothelial cells play a vital role in preserving the integrity of the glomerular filtration barrier [34].

GECs play a crucial role in maintaining the integrity of the glomerular filtration barrier. However, they are susceptible to damage caused by circulating substances such as blood glucose and inflammatory factors. Damage to GECs can disrupt the glomerulus’s permeability, leading to chronic kidney disease (CKD) [35]. Studies have shown that high glucose-induced elevation of inflammatory factors and apoptosis of GECs can result in endothelial damage [36, 37]. Additionally, increased blood glucose levels have been observed to impact kidney hemodynamics in the early stages of diabetic kidney disease (DKD) [38], leading to elevated pressure within the glomerulus [39], which in turn increases the glomerular filtration rate. Prolonged exposure to these conditions can cause damage to endothelial cells, thickening of the basement membrane, and ultimately contribute to the pathological basis of DKD [37, 40]. In our recent study [41], we observed notable effects of TFA in reducing blood glucose levels in rats with DKD. Concurrently, there was a significant reduction in HbA1c levels. These findings indicate the potential beneficial effects of TFA in maintaining stable blood glucose levels.

The development of DKD is strongly influenced by oxidative stress [42]. Oxidative stress has been observed to accelerate the onset and progression of DKD through various pathways [43, 44]. These pathways include the enhancement of glomerular hyperfiltration and direct induction of damage to renal cells. In our current study, we made a significant finding that TFA effectively counteracts oxidative stress in vivo. The administration of TFA successfully inhibited the production of MDA (malondialdehyde) while simultaneously increasing the levels of SOD. Moreover, we observed that TFA significantly preserved the viability of glomerular endothelial cells exposed to high glucose. Additionally, the expression of tight junction proteins in these cells was upregulated. Our study provides compelling evidence that TFA mitigates the development of DKD by preserving the integrity of the glomerular filtration barrier.

Recent studies indicate that inflammatory response, oxidative stress damage, and global filtration barrier abnormalities could contribute to the development and progression of DKD. Mitochondrial dysfunction in DKD leads to impaired ATP production and increased ROS generation, contributing to oxidative stress, inflammation, and cellular damage in the kidneys [45]. Chronic low-grade inflammation occurs in DKD, promoting immune cell recruitment and release of inflammatory molecules, further exacerbating the inflammatory response and damaging the kidneys [46]. Additionally, high glucose levels and mitochondrial dysfunction contribute to increased ROS production, resulting in oxidative stress that promotes inflammation, fibrosis, and cell death in the kidneys [45]. These factors collectively contribute to the pathogenesis of DKD and the disruption of the filtration barrier.

Crucial mechanisms involved in inhibiting the progression of DKD include signaling pathways related to glucose metabolism regulation, antioxidation, anti-inflammation, anti-fibrosis, and podocyte protection. Network pharmacology analysis has revealed that the PI3K/AKT signaling pathway positively impacts DKD by promoting kidney replenishment and dampness removal. Among these, the PI3K/AKT signaling pathway plays a vital role in cell proliferation, growth, and viability [47, 48]. Our present study confirmed that TFA significantly inhibits the phosphorylation of PI3K/AKT, thereby slowing down the progression of DKD. The present study also demonstrated that high glucose-induced degradation of GECs glycocalyx and its core proteins syndecan-1 and glypican-1 decrease in GECs; TFA and metformin up-regulated the expression levels of endothelial glycocalyx, syndecan-1 and glypican-1 in GECs. In addition, our results showed that TFA and metformin decreased high glucose-induced IL-1β and TNF-α release in GECs. Collectively, these results indicated that TFA and metformin could improve high glucose-induced endothelial barrier damage and inflammation in vitro.

**Fig. 9:**
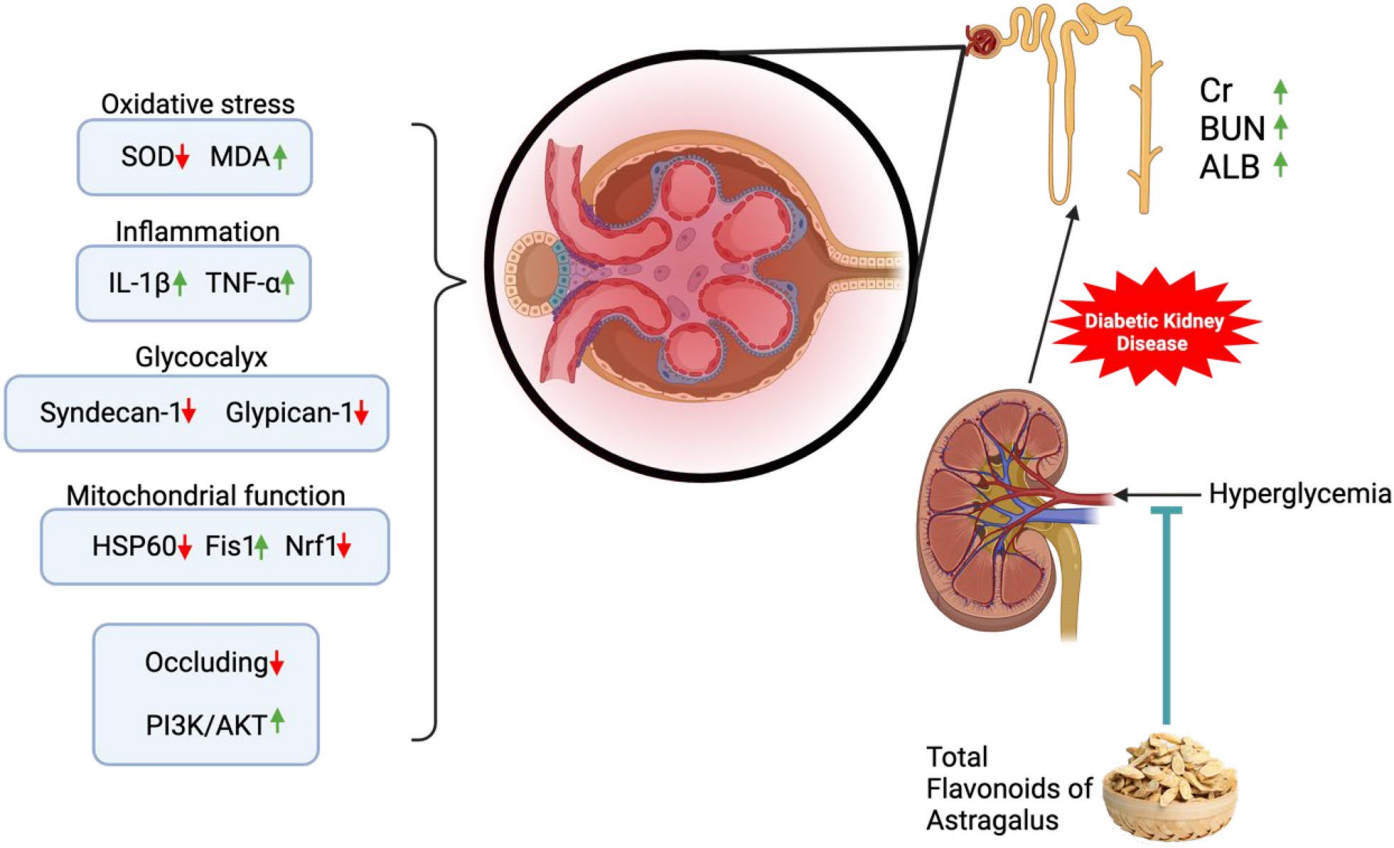
Proposed mechanism of TFA on protecting the glomerular filtration barrier under DKD settings

In summary, our present study demonstrated that TFA can effectively inhibit the progression of DKD by ameliorating renal fibrosis and preserving the integrity of the kidney filtration barrier, and PI3K/AKT pathway plays a crucial role in mediating these effects (Fig. 9). These results provided pharmacological evidence supporting the use of TFA in the treatment of kidney diseases.

### Supplementary Information

**Additional file 1.** Compounds identified by network pharmacologicalanalysis in TFA that could potentially have an active role on diabetic kidney disease.

## Data Availability

All data used in the presented study can get from the corresponding author upon request.

## Abbreviations

ALB: Albumin
BUN: Blood urea nitrogen
CCK-8: Cell counting Kit-8
Cr: Creatinine
DKD: Diabetic kidney disease
GEC: Glomerular endothelial cell
HFD: High fat diet
HG: High glucose
H&E: Hematoxylin–eosin
MDA: Malondialdehyde
NF-κB: Nuclear factor κB
OS: Oxidative stress
ROS: Reactive oxygen species
SOD: Superoxide dismutase
STZ: Streptozotocin
TFA: Total flavonoids of Astragalus
PI3K/AKT: Phosphatidyl-inositol 3-kinase/serine-threonine kinase
UPLC-ESI-Q-TOF–MS/MS: Ultra-performance liquid chromatography coupled to electrospray ionization quadrupole-time of flight mass spectrometry
UPRmt: Mitochondrial unfolded protein response

## References

[1] Dong R, Xu Y (2022). Glomerular cell cross talk in diabetic kidney diseases. J Diabetes.

[2] Navarro JF, Mora C (2005). Role of inflammation in diabetic complications. Nephrol Dial Transpl.

[3] Demircan N, Safran BG, Soylu M, Ozcan AA, Sizmaz S (2006). Determination of vitreous interleukin-1 (IL-1) and tumour necrosis factor (TNF) levels in proliferative diabetic retinopathy. Eye.

[4] Donate-Correa J, Ferri CM, Sánchez-Quintana F, Pérez-Castro A, González-Luis A, Martín-Núñez E (2020). Inflammatory cytokines in diabetic kidney disease: pathophysiologic and therapeutic implications. Front Med.

[5] Jin J, Fang F, Gao W, Chen H, Wen J, Wen X (2021). The structure and function of the glycocalyx and its connection with blood–brain barrier. Front Cell Neurosci.

[6] Qu J, Cheng Y, Wu W, Yuan L, Liu X (2021). Glycocalyx impairment in vascular disease: focus on inflammation. Front Cell Dev Biol.

[7] Mitra R, O'Neil GL, Harding IC, Cheng MJ, Mensah SA, Ebong EE (2017). Glycocalyx in atherosclerosis-relevant endothelium function and as a therapeutic target. Curr Atheroscler Rep.

[8] Maciej-Hulme ML, Van Gemst JJ, Sanderson P, Rops A, Berden JH, Smeets B (2023). Glomerular endothelial glycocalyx-derived heparan sulfate inhibits glomerular leukocyte influx and attenuates experimental glomerulonephritis. Front Mol Biosci.

[9] Li X, Zhao T, Gu J, Wang Z, Lin J, Wang R (2022). Intake of flavonoids from *Astragalus membranaceus* ameliorated brain impairment in diabetic mice via modulating brain-gut axis. Chin Med.

[10] Wang Z, Li XL, Hong KF, Zhao TT, Dong RX, Wang WM (2021). Total flavonoids of Astragalus ameliorated bile acid metabolism dysfunction in diabetes mellitus. Evid Based Complement Alternat Med.

[11] Li J, Xu L, Sang R, Yu Y, Ge B, Zhang X (2018). Immunomodulatory and anti-inflammatory effects of total flavonoids of Astragalus by regulating NF-ΚB and MAPK signalling pathways in RAW 264.7 macrophages. Pharmazie.

[12] Yang L, Han X, Xing F, Wu H, Shi H, Huang F (2021). Total flavonoids of astragalus attenuates experimental autoimmune encephalomyelitis by suppressing the activation and inflammatory responses of microglia via JNK/AKT/NFκB signaling pathway. Phytomedicine.

[13] Hu Q, Qu C, Xiao X, Zhang W, Jiang Y, Wu Z (2021). Flavonoids on diabetic nephropathy: advances and therapeutic opportunities. Chin Med.

[14] Wu Q, Hu Y (2020). Integrated network pharmacology and molecular docking strategy to explore the mechanism of medicinal and edible Astragali Radix-Atractylodis Macrocephalae Rhizoma acting on pneumonia via immunomodulation. J Food Biochem.

[15] Zhang X, Shen T, Zhou X, Tang X, Gao R, Xu L (2020). Network pharmacology based virtual screening of active constituents of *Prunella vulgaris* L. and the molecular mechanism against breast cancer. Sci Rep.

[16] Szklarczyk D, Gable AL, Lyon D, Junge A, Wyder S, Huerta-Cepas J (2019). STRING v11: protein-protein association networks with increased coverage, supporting functional discovery in genome-wide experimental datasets. Nucleic Acids Res.

[17] Younus H (2018). Therapeutic potentials of superoxide dismutase. Int J Health Sci.

[18] Yan Z, Spaulding HR (2020). Extracellular superoxide dismutase, a molecular transducer of health benefits of exercise. Redox Biol.

[19] Yaribeygi H, Atkin SL, Sahebkar A (2019). Interleukin-18 and diabetic nephropathy: a review. J Cell Physiol.

[20] Liew H, Roberts MA, Pope A, McMahon LP (2021). Endothelial glycocalyx damage in kidney disease correlates with uraemic toxins and endothelial dysfunction. BMC Nephrol.

[21] Toda N, Mukoyama M, Yanagita M, Yokoi H (2018). CTGF in kidney fibrosis and glomerulonephritis. Inflamm Regen.

[22] Qiu HY, Fan WR, Huang SM, Liu F, Tang WZ, Zuo C (2010). Effect of high concentration of glucose on thickness of glycocalyx and expression of syndecan-1 and glypican-1 in cultured human renal glomerular endothelial cells. Sichuan Da Xue Xue Bao Yi Xue Ban.

[23] Kuo WT, Shen L, Zuo L, Shashikanth N, Ong M, Wu L (2019). Inflammation-induced occludin downregulation limits epithelial apoptosis by suppressing caspase-3 expression. Gastroenterology.

[24] Xu C, Wu X, Hack BK, Bao L, Cunningham PN (2015). TNF causes changes in glomerular endothelial permeability and morphology through a Rho and myosin light chain kinase-dependent mechanism. Physiol Rep.

[25] Nolden K, Kakarla M, Egner J, Wang J, Harwig M, Puppala V, et al. A novel Fis1 inhibiting peptide reverses diabetic endothelial dysfunction in human resistance arteries. bioRxiv; 2020.

[26] Hu S, Feng J, Wang M, Wufuer R, Liu K, Zhang Z (2022). Nrf1 is an indispensable redox-determining factor for mitochondrial homeostasis by integrating multi-hierarchical regulatory networks. Redox Biol.

[27] Tuttle KR, Wong L, St Peter W, Roberts G, Rangaswami J, Mottl A (2022). Moving from evidence to implementation of breakthrough therapies for diabetic kidney disease. Clin J Am Soc Nephrol.

[28] Tang G, Li S, Zhang C, Chen H, Wang N, Feng Y (2021). Clinical efficacies, underlying mechanisms and molecular targets of Chinese medicines for diabetic nephropathy treatment and management. Acta Pharm Sin B.

[29] Liu Q, Zhang L, Shan Q, Ding Y, Zhang Z, Zhu M (2018). Total flavonoids from Astragalus alleviate endothelial dysfunction by activating the Akt/eNOS pathway. J Int Med Res.

[30] Han R, Wu WQ, Wu XP, Liu CY (2015). Effect of total flavonoids from the seeds of Astragali complanati on natural killer cell function. J Ethnopharmacol.

[31] Heyman SN, Raz I, Dwyer JP, Weinberg Sibony R, Lewis JB, Abassi Z (2022). Diabetic proteinuria revisited: updated physiologic perspectives. Cells.

[32] Li Y, Ji X, Ni W, Luo Y, Ding B, Ma J (2021). Serum albumin and albuminuria predict the progression of chronic kidney disease in patients with newly diagnosed type 2 diabetes: a retrospective study. PeerJ.

[33] Daehn IS, Duffield JS (2021). The glomerular filtration barrier: a structural target for novel kidney therapies. Nat Rev Drug Discov.

[34] Ramnath RD, Satchell SC (2020). Glomerular endothelial cells: assessment of barrier properties in vitro. Methods Mol Biol.

[35] Rico-Fontalvo J, Aroca G, Cabrales J, Daza-Arnedo R, Yánez-Rodríguez T, Martínez-Ávila MC (2022). Molecular mechanisms of diabetic kidney disease. Int J Mol Sci.

[36] Lim JH, Kim HW, Kim MY, Kim TW, Kim EN, Kim Y (2018). Cinacalcet-mediated activation of the CaMKKβ-LKB1-AMPK pathway attenuates diabetic nephropathy in db/db mice by modulation of apoptosis and autophagy. Cell Death Dis.

[37] Gil CL, Hooker E, Larrivée B (2021). Diabetic kidney disease, endothelial damage, and podocyte-endothelial crosstalk. Kidney Med.

[38] Ricciardi CA, Gnudi L (2021). Kidney disease in diabetes: from mechanisms to clinical presentation and treatment strategies. Metabolism.

[39] Fan L, Gao W, Nguyen BV, Jefferson JR, Liu Y, Fan F (2020). Impaired renal hemodynamics and glomerular hyperfiltration contribute to hypertension-induced renal injury. Am J Physiol Renal Physiol.

[40] Zhang J, Wang Y, Gurung P, Wang T, Li L, Zhang R (2018). The relationship between the thickness of glomerular basement membrane and renal outcomes in patients with diabetic nephropathy. Acta Diabetol.

[41] Zhao T, Li M, Xiang Q, Lie B, Chen D, Wang W (2022). Yishen Huashi granules ameliorated the development of diabetic nephropathy by reducing the damage of glomerular filtration barrier. Front Pharmacol.

[42] Piko N, Bevc S, Hojs R, Ekart R (2023). The role of oxidative stress in kidney injury. Antioxidants.

[43] Tanase DM, Gosav EM, Anton MI, Floria M, Seritean Isac PN, Hurjui LL (2022). Oxidative stress and NRF2/KEAP1/ARE pathway in diabetic kidney disease (DKD): new perspectives. Biomolecules.

[44] Ma X, Ma J, Leng T, Yuan Z, Hu T, Liu Q (2023). Advances in oxidative stress in pathogenesis of diabetic kidney disease and efficacy of TCM intervention. Ren Fail.

[45] Galvan DL, Mise K, Danesh FR (2021). Mitochondrial regulation of diabetic kidney disease. Front Med.

[46] Matoba K, Takeda Y, Nagai Y, Kawanami D, Utsunomiya K, Nishimura R (2019). Unraveling the role of inflammation in the pathogenesis of diabetic kidney disease. Int J Mol Sci.

[47] Liu R, Chen Y, Liu G, Li C, Song Y, Cao Z (2020). PI3K/AKT pathway as a key link modulates the multidrug resistance of cancers. Cell Death Dis.

[48] Tian LY, Smit DJ, Jücker M (2023). The role of PI3K/AKT/mTOR signaling in hepatocellular carcinoma metabolism. Int J Mol Sci.

